# The Effect of Age on Performance of the Kidney Failure Risk Equation in Advanced CKD

**DOI:** 10.1016/j.ekir.2021.09.006

**Published:** 2021-10-08

**Authors:** Gregory L. Hundemer, Navdeep Tangri, Manish M. Sood, Edward G. Clark, Mark Canney, Cedric Edwards, Christine A. White, Matthew J. Oliver, Tim Ramsay, Ayub Akbari

**Affiliations:** 1 Division of Nephrology, Department of Medicine, The Ottawa Hospital Research Institute, University of Ottawa, Ottawa, Ontario, Canada; 2 Clinical Epidemiology Program, Ottawa Hospital Research Institute, University of Ottawa, Ottawa, Ontario, Canada; 3 Division of Nephrology, Department of Medicine, University of Manitoba, Winnipeg, Manitoba, Canada; 4 Division of Nephrology, Department of Medicine, Queen’s University, Kingston, Ontario, Canada; 5 Division of Nephrology, Department of Medicine, Sunnybrook Health Sciences Centre, University of Toronto, Toronto, Ontario, Canada

**Keywords:** age, chronic kidney disease, KFRE, kidney failure risk equation, kidney failure, risk prediction

## Abstract

**Introduction:**

The Kidney Failure Risk Equation (KFRE) is a clinical tool widely used to predict progression from chronic kidney disease (CKD) to kidney failure. This study aimed to evaluate the effect of age on KFRE performance in advanced CKD.

**Methods:**

We conducted a retrospective cohort study among 1701 consecutive patients referred to an advanced CKD clinic in Ottawa, Canada, between 2010 and 2018. Patients were categorized by age as follows: <60, 60 to 69, 70 to 79, and ≥80 years. Calibration plots compared the predicted (through the KFRE) and observed incidence of kidney failure. Concordance statistic (C-statistic) evaluated discrimination. Cumulative incidence of kidney failure was compared between models that accounted for the competing risk of death and those that did not.

**Results:**

We found that the KFRE overestimated the risk of kidney failure among the oldest subset of patients (≥80 years) with absolute and relative differences of 7.6% and 22.8%, respectively, over 2 years (*P* = 0.047), and 24.7% and 40.4%, respectively, over 5 years (*P* < 0.001). The degree of overestimation in the elderly was most pronounced among those with the highest predicted risks for kidney failure. KFRE discrimination was acceptable (C-statistic 0.70–0.79) across all age categories. The cumulative incidence of kidney failure was overestimated in models that did not account for the competing risk of death, and this overestimation was more pronounced with older age.

**Conclusion:**

The KFRE overestimates kidney failure risk among elderly patients with advanced CKD. This overestimation relates to the increasing competing risk of death with older age, particularly over longer time horizons.

The global prevalence of CKD is 9.1%, thereby affecting approximately 700 million people, and it continues to rise.^1^ CKD accelerates the risk for kidney failure which carries a high burden of morbidity, mortality, and health care costs.^2^ Although most patients with CKD do not ultimately progress to kidney failure, the proportion of incident CKD cases that will later progress to kidney failure continues to increase.^3^ Therefore, identification of patients with CKD at high risk for progression to kidney failure is essential to provide both targeted treatments attempting to slow CKD progression and advanced care planning regarding kidney replacement therapy modalities, including dialysis and kidney transplantation.

To provide individualized risk estimates for patients with CKD, a number of risk prediction models have been developed and integrated into clinical practice.^4^^,^^5^ The most widely used prediction model, in part related to its simplicity of use, is the KFRE.^6^ The KFRE has been extensively validated in a number of different patient populations and geographic settings.^10^, ^11^, ^12^, ^13^, ^14^, ^6^, ^7^, ^8^, ^9^ The KFRE predicts the risk of an individual patient for kidney failure (defined as dialysis or kidney transplantation) within 2-year and 5-year time frames by incorporating the following variables: age, sex, estimated glomerular filtration rate based on the CKD–Epidemiology Collaboration equation,^15^ and urine albumin-to-creatinine ratio.^6^

Despite widespread external validation, concerns have been raised on the use of risk prediction models, such as the KFRE, in which death before kidney failure is treated as a censoring event rather than a competing event. Not accounting for the competing risk of death has been found to result in an overestimation of the risk of kidney failure particularly over longer periods of follow-up.^5^^,^^16^, ^17^, ^18^ Given the direct relationship between age and mortality, this overestimation of kidney failure risk may be more relevant to elderly populations and less relevant to younger populations—a topic which, to our knowledge, has not been previously studied. Here, we evaluated the impact of age on the 4-variable KFRE performance in a large population of patients with advanced CKD.

## Methods

### Study Design

We performed a retrospective cohort study of adults (≥18 years) with advanced CKD referred to the Ottawa Hospital Multi-Care Kidney Clinic between January 1, 2010, and December 31, 2018. The reporting of this study follows guidelines for prediction model validation (Supplementary Table S1).^19^

### Data Source and Study Cohort

This study was conducted at the Ottawa Hospital Multi-Care Kidney Clinic (Ottawa, Ontario, Canada). The Ottawa Hospital is a 1150-bed academic tertiary care center with a catchment area of approximately 1.3 million people. The Ottawa Hospital Multi-Care Kidney Clinic is a specialty nephrology clinic designed to provide comprehensive, multidisciplinary care for patients with advanced CKD and is the sole such program within the catchment area. Timing of referral is at the discretion of the primary nephrologist, though referrals are suggested when the estimated glomerular filtration rate is <25 ml/min per 1.73 m^2^ or the 2-year KFRE is >20%. Patients are seen in the clinic regularly, from as often as every 2 weeks to a minimum of semiannually, though the exact interval is at the discretion of the nephrologist. At each visit, patients are seen by a nurse, dietician, and nephrologist, with pharmacist and social work support available as needed. Patients are educated on kidney failure treatment options, including hemodialysis, peritoneal dialysis, kidney transplantation, and conservative management.

The study cohort was derived from a database of all patients referred to the Multi-Care Kidney Clinic since January 1, 2010, with follow-up data available to December 31, 2020. The database undergoes routine data audits to ensure accuracy. We included patients initially seen in the clinic between the years 2010 and 2018 (*N* = 1834, Figure 1). From this initial cohort, 133 patients (7%) were lost to follow-up owing to the following: (i) having their nephrology care transferred to a provider outside of the Multi-Care Kidney Clinic, (ii) moving away from the Ottawa area, or (iii) leaving the clinic for another reason. Therefore, we did not have access to outcome data for these patients in regard to kidney failure or death. To evaluate the 5-year KFRE, we included only patients initially seen in the clinic between 2010 and 2015 to ensure at least 5 years of follow-up (*n* = 1176). From this 5-year KFRE cohort, 98 patients (8%) were lost to follow-up owing to the aforementioned reasons listed previously. Ultimately, 1701 patients were included in the data analysis to evaluate the 2-year KFRE whereas 1078 patients were included in the data analysis to evaluate the 5-year KFRE. Patients were subclassified by age categories within the study cohort, such as follows: <60 years, 60 to 69 years, 70 to 79 years, and ≥80 years. All protocols were approved by the Ottawa Health Science Network Research Ethics Board (protocol identification 20200506-01H). Informed consent requirements were waived owing to the retrospective nature of the data.

**Figure 1:**
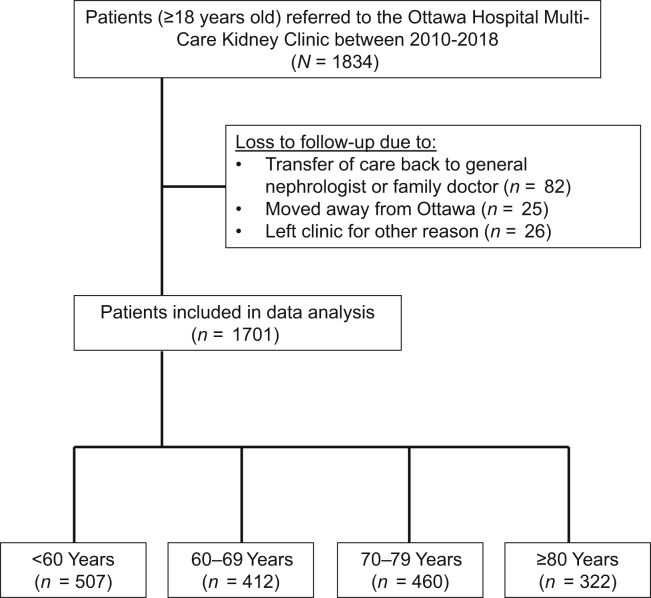
Study flow diagram.

### Outcome

The outcome of interest was the observed incidence of kidney failure (dialysis or kidney transplantation) within 2 and 5 years from initial clinic referral.

### Predictors

The predictors used in this study were the 2-year and 5-year 4-variable KFRE scores (North America calibrated) at the time of initial clinic referral (see the Supplementary Material for the full equations).^7^

### Statistical Analysis

For baseline data, continuous variables were expressed as mean (SD) if normally distributed and as median (25th–75th percentile interquartile range) if non-normally distributed, whereas categorical variables were expressed as numbers (%). The variables necessary to calculate the KFRE were collected at the initial visit as per clinic protocol; therefore, these data were available for the entire cohort with no missing data. Urine albumin-to-creatinine ratio was obtained from a random spot sample. If a patient died before kidney failure, the patient was included in the analysis as not having developed kidney failure within that time frame. Death was not treated as a competing risk in our primary analyses so as to align with the design of the original KFRE derivation^6^; however, an additional analysis accounting for death as a competing risk was included.

#### Predicted Versus Observed Risks of Kidney Failure

Predicted (based on the KFRE) versus observed risks of kidney failure were compared at the 2-year and 5-year time points across age categories. Fisher exact testing was used to test for statistically significant differences between predicted and observed incidences of kidney failure. We chose this approach to evaluate KFRE performance using actual (rather than modeled) observed kidney failure incidence within 2 distinct time windows (i.e., 2 and 5 years) so as to mirror how the KFRE is applied in real-word clinical practice.

#### Discrimination

Discrimination refers to the ability of a model to separate individuals with and without disease. We used time-to-event (Cox) models to measure the C-statistic (95% CI) for the 2-year and 5-year KFRE for the total population and across age categories. We defined the quality of discrimination based on the C-statistic as follows: ≥0.90 = outstanding, 0.80 to 0.89 = excellent, 0.70 to 0.79 = acceptable, 0.51 to 0.69 = poor, and 0.50 = no discrimination.^20^

#### Calibration

Calibration refers to the agreement between predicted and observed risks. We used loess smoothing curves to compare the observed incidence of kidney failure (along with the 95% CI) to the predicted incidence of kidney failure within both the 2-year and 5-year time points across age categories.

#### Assessment of Death as a Competing Risk

In the presence of competing risks (such as death), Kaplan–Meier curves are known to overestimate risk.^21^ To evaluate the effect of the competing risk of death before kidney failure as a function of age, we performed survival analyses across age categories comparing the Kaplan–Meier curve (where death before kidney failure was censored) with the cumulative incidence function determined using Fine and Gray models (where death before kidney failure was treated as a competing risk).

All statistical analyses were performed using SAS version 9.4. All *P* values are 2-sided with values < 0.05 considered significant.

## Results

### Patient Characteristics

There were 1701 patients who were included in the 2-year KFRE analysis (Table 1), whereas 1078 patients were included in the 5-year KFRE analysis (Supplementary Table S2). Furthermore, 507 patients (30%) were <60 years of age, 412 patients (24%) were 60 to 69 years of age, 460 patients (27%) were 70 to 79 years of age, and 322 patients (19%) were ≥80 years of age. Most of the population was male (1052 of 1701 [62%]) and of White race (1256 of 1701 [74%]). The mean (SD) estimated glomerular filtration rate among the total population was 17 (6) ml/min per 1.73 m^2^ and similar across age categories. The median (interquartile range) urine albumin-to-creatinine ratio was 1389 (407–2938) mg/g with generally lower levels of albuminuria in the higher age categories. For the 2-year KFRE cohort, the median 2-year KFRE score was 41% (interquartile range 22%–64%) (Table 1) and decreased with age. For the 5-year KFRE cohort, the median baseline 5-year KFRE score was 81% (interquartile range 51%–96%) (Supplementary Table S2) and decreased with age. Supplementary Table S3 compares the original KFRE development cohort^6^ with the current validation cohort.

**Table 1:** Baseline characteristics of 1701 patients with advanced CKD referred to the Ottawa Hospital Multi-Care Kidney Clinic from 2010 to 2018

Baseline characteristics	Total population *N* = 1701	<60 yr *n* = 507	60–69 yr *n* = 412	70–79 yr *n* = 460	≥80 yr *n* = 322
Demographics					
Age, yr, mean (SD)	66 (15)	48 (10)	65 (3)	75 (3)	85 (4)
Female, *n* (%)	649 (38)	194 (38)	151 (37)	171 (37)	133 (41)
Race, *n* (%)					
White	1256 (74)	336 (66)	312 (76)	352 (77)	256 (80)
Black	76 (4)	38 (7)	23 (6)	9 (2)	6 (2)
Asian	89 (5)	25 (5)	16 (4)	24 (5)	24 (7)
Other/unknown	280 (16)	108 (21)	61 (15)	75 (16)	36 (11)
Baseline kidney parameters					
Serum creatinine mg/dl, mean (SD)	3.5 (1.1)	3.7 (1.3)	3.6 (1.1)	3.3 (0.9)	3.3 (0.9)
eGFR ml/min per 1.73 m^2^, mean (SD)	17 (6)	19 (7)	17 (6)	17 (5)	16 (5)
Urine albumin-to-creatinine ratio mg/g, median (IQR)	1389 (407–2938)	1645 (672–3290)	1698 (547–3142)	1209 (362–2643)	781 (204–2231)
Other laboratory data					
Serum potassium mEq/l, mean (SD)	4.5 (0.7)	4.6 (0.8)	4.5 (0.6)	4.5 (0.6)	4.5 (0.6)
Serum calcium mg/dl, mean (SD)	8.9 (0.6)	8.9 (0.6)	8.9 (0.6)	9.0 (0.6)	8.9 (0.6)
Serum phosphate mg/dl, mean (SD)	4.2 (0.9)	4.2 (1.0)	4.3 (1.0)	4.2 (0.8)	4.2 (0.9)
Serum bicarbonate mEq/l, mean (SD)	24 (3)	24 (3)	24 (4)	24 (3)	24 (4)
Serum albumin g/dl, mean (SD)	3.5 (0.5)	3.5 (0.5)	3.4 (0.5)	3.5 (0.4)	3.5 (0.5)
Blood pressure data					
Systolic blood pressure mm Hg, mean (SD)	137 (20)	136 (20)	136 (20)	137 (19)	138 (20)
Diastolic blood pressure mm Hg, mean (SD)	71 (13)	79 (13)	71 (12)	68 (11)	65 (10)
ACE inhibitor/ARB use*, n* (%)	847 (50)	297 (59)	192 (47)	227 (49)	131 (41)
Diuretic, *n* (%)	1050 (62)	254 (50)	279 (68)	320 (70)	197 (61)
Body mass index, kg/m^2^, mean (SD)	29.8 (6.9)	29.9 (7.9)	31.2 (7.3)	30.0 (6.1)	27.5 (5.2)
Diabetes mellitus*, n* (%)	1023 (60)	244 (48)	295 (72)	314 (68)	170 (53)
KFRE, %					
2-yr KFRE					
Mean (SD)	44 (25)	53 (27)	47 (25)	39 (22)	33 (21)
Median (IQR)	41 (22–64)	54 (30–76)	45 (27–68)	37 (20–56)	28 (17–49)
5-yr KFRE					
Mean (SD)	73 (25)	80 (24)	76 (23)	70 (25)	63 (26)
Median (IQR)	81 (55–96)	91 (67–99)	84 (63–97)	76 (50–92)	64 (43–88)

ACE, angiotensin-converting enzyme; ARB, angiotensin II receptor blocker; CKD, chronic kidney disease; eGFR, estimated glomerular filtration rate; IQR, interquartile range; KFRE, kidney failure risk equation.

### Predicted Versus Observed Risks of Kidney Failure

**Figure 2:**
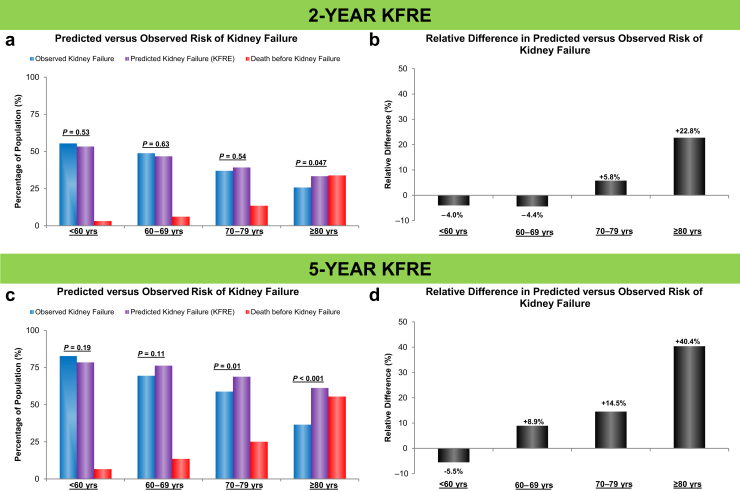
Predicted versus observed rates of kidney failure and rates of death before kidney failure by age. (a) Predicted versus observed risks of kidney failure and occurrence of death before kidney failure for the 2-year KFRE across age categories; (b) relative difference in predicted versus observed risks of kidney failure [(predicted − observed) / predicted] for the 2-year KFRE across age categories; (c) predicted versus observed risks of kidney failure and occurrence of death before kidney failure for the 5-year KFRE across age categories; and (d) relative difference in predicted versus observed risks of kidney failure [(predicted − observed) / predicted] for the 5-year KFRE across age categories. *P* values in a and c represent Fisher exact test results comparing predicted versus observed risks of kidney failure. KFRE, kidney failure risk equation; yrs, years.

Table 2 displays the observed rates of kidney failure and death before kidney failure. The differences between predicted (through the KFRE) versus observed risks of kidney failure at 2 and 5 years by age category are displayed in Figure 2.

**Table 2:** Observed rates of kidney failure and death before kidney failure for the 2-yr and 5-yr KFRE analyses

Observed rates of kidney failure and death before kidney failure	Total population	<60 yr	60–69 yr	70–79 yr	≥80 yr
2-yr analysis					
Total population, *n*	1701	507	412	460	322
Kidney failure, *n* (%)	735 (43)	281 (55)	201 (49)	170 (37)	83 (26)
Deaths before kidney failure*, n* (%)	212 (12)	16 (3)	25 (6)	62 (13)	109 (34)
5-yr analysis					
Total population, *n*	1078	319	252	296	211
Kidney failure*, n* (%)	690 (64)	264 (83)	175 (69)	174 (59)	77 (36)
Deaths before kidney failure, *n* (%)	246 (23)	21 (7)	34 (13)	74 (25)	117 (55)

KFRE, kidney failure risk equation.

For the 2-year KFRE, there was no statistically significant difference between the predicted and observed risks for the 3 youngest age categories (Figure 2a and b). Nevertheless, there was a significant difference with the oldest age category (≥80 years) wherein the KFRE overestimated the risk of kidney failure by an absolute difference of 7.6% (*P* = 0.047) corresponding to a relative difference in risk (i.e., absolute risk difference ÷ predicted risk) of 22.8%. Notably, the rate of death before kidney failure had a direct relationship with age and exceeded the observed rate of kidney failure in the oldest age category.

For the 5-year KFRE, there was no statistically significant difference between the predicted and observed risks for the 2 youngest age categories (Figure 2c and d). Nevertheless, there was a significant difference with the 2 oldest age categories (70–79 years and ≥80 years) with the KFRE overestimating the risk of kidney failure by absolute differences of 10.0% (*P* = 0.01) and 24.7% (*P* < 0.001) and relative differences of 14.5% and 40.4%, respectively. Again, the rate of death before kidney failure had a direct relationship with age and exceeded the observed rate of kidney failure in the oldest age category.

### Discrimination

Table 3 displays the C-statistic values based on time-to-event analyses using the 2-year and 5-year KFRE across age categories. The KFRE displayed acceptable discrimination (C-statistic 0.70–0.79)^20^ for the total population and across all age categories within both the 2-year and 5-year time frames.

**Table 3:** C-Statistic (95% CI) for the 2-year and 5-year KFRE by age category

C-Statistic (95% CI) for the KFRE by age category		
Age category	2-yr KFRE	5-yr KFRE
Total population	0.77 (0.75–0.79)	0.76 (0.74–0.78)
<60 yr	0.77 (0.74–0.80)	0.75 (0.72–0.78)
60–69 yr	0.77 (0.73–0.80)	0.77 (0.74–0.80)
70–79 yr	0.76 (0.71–0.81)	0.75 (0.71–0.80)
≥80 yr	0.73 (0.65–0.80)	0.76 (0.68–0.84)

C-statistic, concordance statistic; KFRE, kidney failure risk equation.

### Calibration

**Figure 3:**
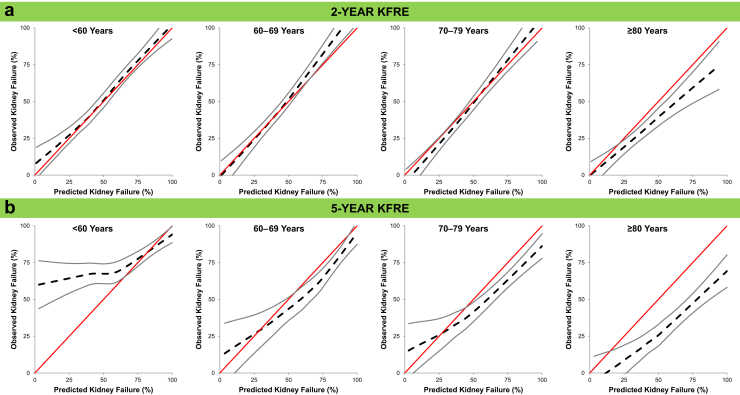
Calibration plots for the (a) 2-year and (b) 5-year KFRE by age. Plots are displayed as loess smoothing curves. Red lines are displayed as a reference to represent perfect calibration (where predicted risk exactly equals observed risk). Dashed black lines represent the actual relationships between predicted and observed risks within the cohort. Gray lines represent the 95% CIs for the observed risk of kidney failure. KFRE, kidney failure risk equation.

Figure 3 displays the calibration plots comparing the predicted versus observed risks of kidney failure by age categories with the 2-year and 5-year KFRE. For the 2-year KFRE, calibration was good across all age categories with the exception of the oldest age category (≥80 years) wherein there was greater risk overestimation among patients with higher predicted risks for kidney failure based on the KFRE (Figure 3a).

For the 5-year KFRE, there was risk overestimation based on the KFRE across the 3 oldest age categories (60–69 years, 70–79 years, and ≥80 years) with a greater degree of overestimation among those of older age and higher predicted risks for kidney failure (Figure 3b). Notably, among the youngest age group (<60 years), the KFRE underestimated the risk of kidney failure among patients with a lower predicted risk for kidney failure.

### Assessment of Death as a Competing Risk

**Figure 4:**
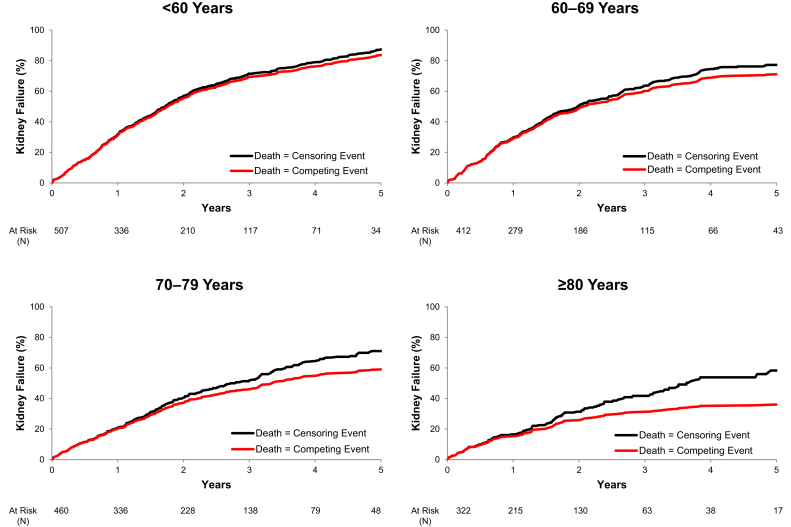
The impact of the competing risk of death on kidney failure incidence by age. Black lines represent one minus the Kaplan–Meier estimate which treats death before kidney failure is treated as a censoring event (correlating with how the KFRE treats death). Red lines represent the cumulative incidence function which treats death before kidney failure as a competing event. KFRE, kidney failure risk equation.

Figure 4 displays the results of an additional analysis comparing the probability of kidney failure by age category within our 2-year and 5-year cohorts, respectively, using one minus the Kaplan–Meier estimate (death before kidney failure treated as a censoring event) versus the cumulative incidence function (death before kidney failure treated as a competing event). These results revealed an overestimation of the cumulative incidence of kidney failure when not accounting for the competing risk of death (as the KFRE was designed)^6^ that increases with advancing age.

## Discussion

Although the KFRE overall performed well in regard to calibration and discrimination in this advanced CKD cohort, we found that the KFRE overestimated the risk of kidney failure among older patients (particularly those ≥80 years of age). This overestimation in the risk of kidney failure by the KFRE relates to the magnified competing risk of death with older age and increases over longer time horizons.

Our study expands on previous work evaluating the impact of the competing risk of death on the performance of kidney failure risk prediction models. Ravani *et al.*^17^ analyzed a large population of patients with stage 4 CKD to evaluate the influence of treating death before kidney failure as a censoring event versus a competing event in the risk prediction of kidney failure. Prediction models with death treated as a censoring event provided kidney failure risk estimates that were 7% higher at 5 years and 19% higher at 10 years compared with models with death treated as a competing event.^17^ In an extension of these findings, a recent study by Ramspek *et al.*^5^ directly compared the predictive performance of 11 kidney failure risk prediction models. Although the KFRE performed well within 2 years, it overestimated the risk of kidney failure over 5 years owing to the competing risk of death.^5^ Alternative risk prediction models that accounted for the competing risk of death, such as the Grams model,^22^ were better suited to estimate kidney failure over longer time frames.^5^

Our results now highlight that the overestimation of risk by the KFRE in advanced CKD is predominately driven by the oldest subset of patients. For the oldest patients within our study cohort (age ≥ 80 years), the KFRE overestimated the risk of kidney failure by absolute differences of 7.6% and 24.7% and relative differences of 22.8% and 40.4% at the 2-year and 5-year time frames, respectively. Therefore, the KFRE provides overly pessimistic risk predictions in regard to kidney failure primarily for the elderly population with advanced CKD who are at high risk for both kidney failure and death. This overestimation was most prominent among the elderly population at the highest risk for kidney failure (i.e., those with the highest KFRE scores).

Is overestimation of kidney failure risk a problem? Certainly, risk overestimation may be preferable to risk underestimation which could lead to lack of preparation for impending kidney failure and subsequent “crash” dialysis starts. Also, if the degree of risk overestimation is small (e.g., <5%), this may not be enough of a difference to sway clinical decision-making on an individual level. Nevertheless, the more pronounced risk overestimation found with the KFRE in the elderly population with advanced CKD may have important implications surrounding both patient counseling and population-level resource allocation related to kidney failure. In elderly individuals who are wishing to undergo future dialysis, if required, it would allow for planning and investigations to be undertaken with the understanding that predialysis mortality remains a significant risk that may approach or even surpass that of needing kidney replacement therapy. As such, they may choose to delay invasive procedures or interventions until they are more imminently required. In addition, countries such as Canada use the KFRE as a policy-level tool to allocate predialysis resources to patient populations most in need.^23^ Acknowledging these limitations in KFRE performance in the elderly population with advanced CKD may affect future policy decisions to better direct these resources to those who may benefit the most from them.

Our study has several notable strengths. First, it included a large continuous sample of patients with advanced CKD with a high event rate. Second, given the clinic protocol for laboratory data collected at the initial referral visit, we were able to determine the KFRE for all patients seen in our clinic throughout the study period. Third, as the Ottawa Hospital Multi-Care Kidney Clinic is the only such clinic within the catchment area, the vast majority of patients (>90%) continued to be followed in the clinic until the end of the prespecified study period, kidney failure, or death, thereby minimizing loss to follow-up.

We acknowledge several limitations. First, this was a single-center study with a predominantly White population which may limit the generalizability of the findings to other clinical settings and racial demographics. This may be particularly relevant for Black patients given the growing debate on racial disparities surrounding most often used estimated glomerular filtration rate equations which may underestimate the severity of kidney disease in the Black population.^24^ Certainly, the impact this may have on kidney failure risk prediction models is an important area which needs to be explored in the future; however, we were unable to address this given the small number of Black patients in our study population. Second, our study cohort had a higher percentage of patients with diabetes mellitus (and likely diabetic kidney disease) compared with previous demographic data on the population with advanced CKD across Canada.^25^^,^^26^ This difference may relate to the referral process for the Multi-Care Kidney Clinic which is at the discretion of the primary nephrologist and may lead to selection bias in terms of which patients enter the clinic which may differ from the population with advanced CKD at large. Finally, the study population size for our assessment of the 5-year KFRE was more restricted than that of the 2-year KFRE to allow patients the necessary follow-up time needed to evaluate model performance. This may have limited our ability to detect differences in 5-year KFRE performance as compared with 2-year KFRE performance.

In conclusion, although the KFRE overall performs well in the advanced CKD setting, it overestimates the risk of kidney failure among elderly patients with advanced CKD. This overestimation of kidney failure risk by the KFRE is related to the competing risk of death which becomes more prominent with advanced age and longer prediction horizons. Nephrologists should be aware of this limitation of the KFRE as it may affect advanced care planning for the elderly population with advanced CKD.

## Disclosure

MMS reports receiving speaker fees from AstraZeneca. MJO is a contracted Medical Lead at Ontario Renal Network and Ontario Health, owner of Oliver Medical Management Inc., which licenses Dialysis Management Analysis and Reporting System software, and has received honoraria for speaking from Baxter Healthcare and participating on advisory boards for Janssen and Amgen. All other authors declared no competing interests.

## Supplementary Material

Supplementary File (PDF)

**2-Year and 5-Year 4-Variable KFRE Equations (North America Calibrated).**

**Table S1.** TRIPOD checklist for prediction model validation.

**Table S2.** Baseline characteristics of the 5-year KFRE cohort.

**Table S3.** Comparison of study cohort to the KFRE development cohort.

**STROBE Statement** (PDF)
