# Supplementary material for: The Effect of Age on Performance of the Kidney Failure Risk Equation in Advanced CKD
Source: Kidney Int Rep. 2021 Oct 8;6(12):2993–3001. doi: 10.1016/j.ekir.2021.09.006 (PMC8640561; doi:10.1016/j.ekir.2021.09.006)
Supplement: Supplementary File (PDF) [file mmc1.pdf]

<u>Supplementary Material</u>

The Effect of Age on Performance of the Kidney Failure Risk Equation in Advanced CKD

Pages

2-Year and 5-Year 4-Variable KFRE Equations (North America Calibrated) 2

Table S1. TRIPOD checklist for prediction model validation. 3-4

Table S2. Baseline characteristics of the 5-year KFRE cohort. 5-6

Table S3. Comparison of study cohort to the KFRE development cohort. 7

References 8

2-Year and 5-Year 4-Variable KFRE Equations (North America Calibrated)[1]

2-Year Equation: 1 – 0.9751 ^ exp (-0.2201 × (age/10 – 7.036) + 0.2467 × (male – 0.5642) – 0.5567 × (eGFR/5 – 7.222) + 0.4510 × (logACR – 5.137))

5-Year Equation: 1 – 0.8996 ^ exp (-0.2201 × (age/10 – 7.036) + 0.2467 × (male – 0.5642) – 0.5567 × (eGFR/5 – 7.222) + 0.4510 × (logACR – 5.137))

Table S1. TRIPOD checklist for prediction model validation.

Section/Topic	[illegible]	Checklist Item	Page
**Title and abstract**			
Title	1	Identify the study as developing and/or validating a multivariable prediction model, the target population, and the outcome to be predicted.	1
Abstract	2	Provide a summary of objectives, study design, setting, participants, sample size, predictors, outcome, statistical analysis, results, and conclusions.	2-3
**Introduction**			
Background and objectives	3a	Explain the medical context (including whether diagnostic or prognostic) and rationale for developing or validating the multivariable prediction model, including references to existing models.	4-5
	3b	Specify the objectives, including whether the study describes the development or validation of the model or both.	5
**Methods**			
Source of data	4a	Describe the study design or source of data (e.g., randomized trial, cohort, or registry data), separately for the development and validation data sets, if applicable.	6-7
	4b	Specify the key study dates, including start of accrual; end of accrual; and, if applicable, end of follow-up.	6-7
Participants	5a	Specify key elements of the study setting (e.g., primary care, secondary care, general population) including number and location of centres.	6-7
	5b	Describe eligibility criteria for participants.	6-7
	5c	Give details of treatments received, if relevant.	N/A
Outcome	6a	Clearly define the outcome that is predicted by the prediction model, including how and when assessed.	7
	6b	Report any actions to blind assessment of the outcome to be predicted.	N/A
Predictors	7a	Clearly define all predictors used in developing or validating the multivariable prediction model, including how and when they were measured.	8
	7b	Report any actions to blind assessment of predictors for the outcome and other predictors.	N/A
Sample size	8	Explain how the study size was arrived at.	7
Missing data	9	Describe how missing data were handled (e.g., complete-case analysis, single imputation, multiple imputation) with details of any imputation method.	7-8
Statistical analysis methods	10c	For validation, describe how the predictions were calculated.	8
	10d	Specify all measures used to assess model performance and, if relevant, to compare multiple models.	8-10
	10e	Describe any model updating (e.g., recalibration) arising from the validation, if done.	N/A
Risk groups	11	Provide details on how risk groups were created, if done.	7
Development vs. validation	12	For validation, identify any differences from the development data in setting, eligibility criteria, outcome, and predictors.	Table S3
**Results**			
Participants	13a	Describe the flow of participants through the study, including the number of participants with and without the outcome and, if applicable, a summary of the follow-up time. A diagram may be helpful.	11, Fig 1
	13b	Describe the characteristics of the participants (basic demographics, clinical features, available predictors), including the number of participants with missing data for predictors and outcome.	11, Tab 1
	13c	For validation, show a comparison with the development data of the distribution of important variables (demographics, predictors and outcome).	Table S3
Model performance	16	Report performance measures (with CIs) for the prediction model.	11-14
Model-updating	17	If done, report the results from any model updating (i.e., model specification, model performance).	N/A
**Discussion**			
Limitations	18	Discuss any limitations of the study (such as nonrepresentative sample, few events per predictor, missing data).	17-18
Interpretation	19a	For validation, discuss the results with reference to performance in the development data, and any other validation data.	15-18
	19b	Give an overall interpretation of the results, considering objectives, limitations, results from similar studies, and other relevant evidence.	15-18

Implications	20	Discuss the potential clinical use of the model and implications for future research.	15-18
**Other information**			
Supplementary information	21	Provide information about the availability of supplementary resources, such as study protocol, Web calculator, and data sets.	Supplementary Material
Funding	22	Give the source of funding and the role of the funders for the present study.	Title Page

Table S2. Baseline characteristics of the 5-year KFRE cohort.

Baseline Characteristics	Total Population N=1078	<60 Years N=319	60-69 Years N=252	70-79 Years N=296	≥80 Years N=211
**Demographics**					
Age, yr, mean (SD)	67 (15)	49 (10)	65 (3)	75 (3)	85 (4)
Female, N (%)	423 (39)	122 (38)	95 (38)	112 (38)	94 (45)
Race, N (%)					
White	808 (75)	224 (70)	192 (76)	227 (77)	165 (78)
Black	59 (5)	32 (10)	14 (6)	7 (2)	6 (3)
Asian	58 (5)	12 (4)	10 (4)	17 (6)	19 (9)
Other/Unknown	153 (14)	51 (16)	36 (14)	45 (15)	21 (10)
**Baseline Kidney Parameters**					
Serum Creatinine mg/dL, mean (SD)	3.5 (1.1)	3.7 (1.3)	3.6 (1.2)	3.4 (0.9)	3.3 (1.0)
eGFR mL/min/1.73m^2, mean (SD)	17 (6)	19 (7)	17 (6)	17 (5)	16 (5)
Urine Albumin-to-Creatinine Ratio mg/g, median (IQR)	1266 (319, 2912)	1576 (460, 3384)	1599 (437, 3177)	1104 (274, 2460)	677 (167, 2071)
**Other Laboratory Data**					
Serum Potassium mEq/L, mean (SD)	4.5 (0.7)	4.6 (0.9)	4.4 (0.6)	4.4 (0.6)	4.4 (0.6)
Serum Calcium mg/dL, mean (SD)	8.9 (0.6)	8.8 (0.6)	8.8 (0.6)	9.0 (0.5)	8.9 (0.6)
Serum Phosphate mg/dL, mean (SD)	4.2 (0.9)	4.2 (1.0)	4.2 (1.0)	4.1 (0.8)	4.2 (0.9)
Serum Bicarbonate mEq/L, mean (SD)	24 (3)	23 (3)	24 (4)	24 (3)	24 (4)
Serum Albumin g/dL, mean (SD)	3.5 (0.5)	3.5 (0.5)	3.4 (0.5)	3.5 (0.4)	3.5 (0.4)
**Blood Pressure Data**					
Systolic Blood Pressure mmHg, mean (SD)	136 (19)	135 (19)	135 (18)	136 (19)	138 (21)
Diastolic Blood Pressure mmHg, mean (SD)	70 (12)	77 (13)	70 (12)	67 (11)	64 (11)

ACE Inhibitor/ARB Use, N (%)	549 (51)	188 (59)	125 (50)	146 (49)	90 (43)
Diuretic, N (%)	679 (63)	165 (52)	170 (67)	209 (71)	135 (64)
**Body Mass Index, kg/m^2, Mean (SD)**	29.8 (6.8)	29.7 (7.5)	31.3 (7.2)	30.0 (6.1)	27.7 (5.4)
**Diabetes Mellitus, N (%)**	649 (60)	159 (50)	179 (71)	201 (68)	110 (52)
**KFRE, %**					
2-Year KFRE					
Mean (SD)	44 (26)	52 (28)	47 (25)	39 (23)	33 (22)
Median (IQR)	41 (21, 65)	53 (26, 77)	45 (27, 68)	36 (18, 56)	27 (15, 49)
5-Year KFRE					
Mean (SD)	72 (27)	78 (26)	76 (24)	69 (26)	61 (28)
Median (IQR)	81 (51, 96)	91 (62, 99)	85 (63, 97)	76 (46, 92)	63 (41, 88)

Abbreviations: ACE, angiotensin-converting enzyme; ARB, angiotensin II receptor blocker; dL, deciliter; eGFR, estimated glomerular filtration rate; g, gram; IQR, interquartile range; KFRE, kidney failure risk equation; kg, kilogram; m, meter; mEq, milliequivalents; mg, milligram; mL, milliliter; mmHg, millimeters of mercury; min, minute; N, number; SD, standard deviation; yr, year.

Table S3. Comparison of study cohort to the KFRE development cohort.[2]

<u>Comparison of Study Validation Cohort to the KFRE Development Cohort</u>

	Current Study Cohort (N=1701)	Development Cohort (N=3449)
Age, yr, mean (SD)	66 (15)	70 (14)
Female sex, N (%)	649 (38)	1503 (44)
Mean systolic BP, mmHg, mean (SD)	137 (20)	130 (22)
Mean diastolic BP, mmHg, mean (SD)	71 (13)	71 (12)
Baseline eGFR, mL/min/1.73m^2, mean (SD)	17 (6)	36 (13)
Serum creatinine, mg/dL, mean (SD)	3.5 (1.1)	2.2 (1.3)
Serum calcium, mg/dL, mean (SD)	8.9 (0.6)	9.4 (0.6)
Serum phosphate, mg/dL, mean (SD)	4.2 (0.9)	4.0 (0.9)
Serum albumin, mg/dL, mean (SD)	3.5 (0.5)	4.0 (0.5)
Serum bicarbonate, mEq/L, mean (SD)	24 (3)	26 (4)
Urine albumin-to-creatinine ratio, mg/g, median (IQR)	1389 (2531)	93 (378)
Kidney failure events	735 (43)	386 (11)

Abbreviations: BP, blood pressure; dL, deciliter; g, gram; IQR, interquartile range; KFRE, kidney failure risk equation; m, meter; mEq, milliequivalents; mg, milligram; mL, milliliter; mmHg, millimeters of mercury; min, minute; N, number; SD, standard deviation; yr, year.

REFERENCES

1. Tangri N, Grams ME, Levey AS*, et al.* Multinational Assessment of Accuracy of Equations for Predicting Risk of Kidney Failure: A Meta-analysis. *JAMA* 2016; **315:** 164-174.

2. Tangri N, Stevens LA, Griffith J*, et al.* A predictive model for progression of chronic kidney disease to kidney failure. *JAMA* 2011; **305:** 1553-1559.
